# Association of *VEGF* haplotypes with breast cancer risk in North-West Indians

**DOI:** 10.1186/s12920-021-01060-4

**Published:** 2021-08-24

**Authors:** Vasudha Sambyal, Kamlesh Guleria, Ruhi Kapahi, Mridu Manjari, Meena Sudan, Manjit Singh Uppal, Neeti Rajan Singh

**Affiliations:** 1 Human Cytogenetics Laboratory, Department of Human Genetics, Guru Nanak Dev University, Amritsar, 143005 Punjab India; 2 Department of Pathology, Sri Guru Ram Das Institute of Medical Sciences and Research, Amritsar, Punjab India; 3 Department of Radiotherapy, Sri Guru Ram Das Institute of Medical Sciences and Research, Amritsar, Punjab India; 4 Department of Surgery, Sri Guru Ram Das Institute of Medical Sciences and Research, Amritsar, Punjab India

**Keywords:** *VEGF*, Polymorphism, Haplotype, Breast cancer

## Abstract

**Background:**

Angiogenesis is a complex and coordinated process regulated by different growth factors and is one of the hallmark features of cancer. VEGF is one of the most important endothelial cell mitogen and has a critical role in normal physiological and tumor angiogenesis. The objective of this study was to investigate the potential association of haplotypes of six *VEGF* polymorphisms with breast cancer risk in North-West Indians.

**Methods:**

Samples of 250 breast cancer patients and 250 age and sex matched controls were genotyped for *VEGF* −2578C/A, −2549I/D, −460T/C, +405C/G, −7C/T and +936C/T polymorphisms. Haplotypes were generated to determine the better contribution of *VEGF* polymorphisms to breast cancer risk.

**Results:**

Haplotypes CDTCCC (OR = 0.56, 95%CI, 0.38–0.81; *p* = 0.003) and CDTGCC (OR = 0.63, 95%CI, 0.44–0.92; *p* = 0.018) of *VEGF* −2578C/A, −2549I/D, −460T/C, +405C/G, −7C/T and +936C/T polymorphisms were significantly associated with decreased risk of breast cancer. CDTCCC haplotype was also significantly associated with reduced risk of breast cancer in pre and post menopausal as well as both obese and non obese patients. Haplotype CDTGCC was marginally associated (*p* = 0.07) with reduced risk of breast cancer in non-obese patients as compared with non-obese controls where as haplotype AICGTC was marginally associated (*p* = 0.09) with reduced risk of breast cancer in obese patients when compared with non-obese patients. The CDTGCC haplotype was significantly associated with increased risk of breast cancer in premenopausal obese patients (OR = 1.98, 95%CI, 1.10–3.56; *p* = 0.02).

**Conclusions:**

Our data indicated that CDTCCC and CDTGCC haplotypes of *VEGF* −2578C/A, −2549I/D, −460T/C, +405C/G, −7C/T and +936C/T polymorphisms were significantly associated with breast cancer risk in North-West Indians. Further studies on multiethnic groups with larger sample size are required to confirm our results.

**Supplementary Information:**

The online version contains supplementary material available at 10.1186/s12920-021-01060-4.

## Background

Angiogenesis is one of the hallmark features of cancer [1]. It is a complex and coordinated process regulated by different growth factors like platelet derived growth factor, transforming growth factor and angiopoietins among which vascular endothelial growth factors (VEGF) play a crucial role [2–4]. VEGF is one of the most powerful endothelial cell mitogen and has a very critical role in normal physiological and tumor angiogenesis [5–7]. It enhances tumor vessel permeability and endothelial cell proliferation, migration, differentiation, capillary formation and also has proinflammatory actions [8–12].

**Fig. 1:**
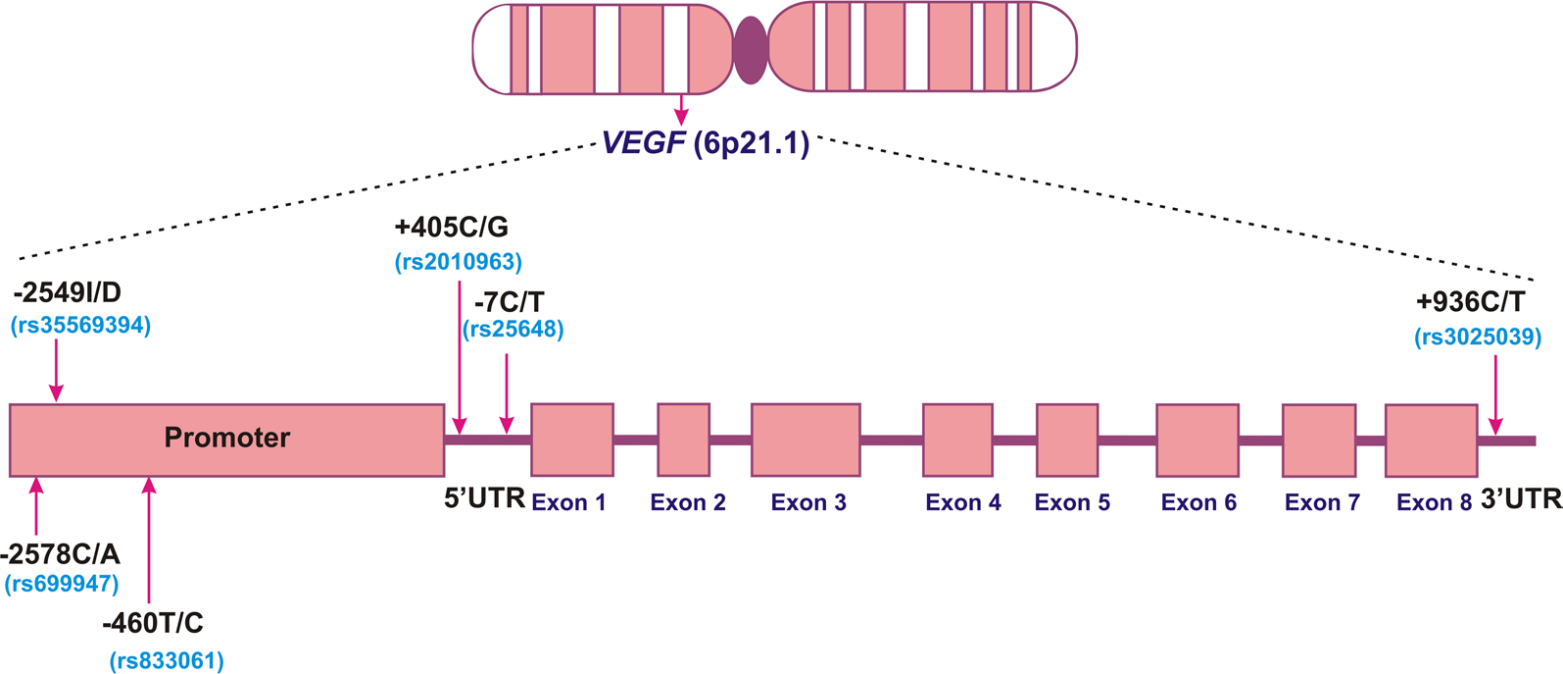
Schematic diagram showing chromosomal position of *VEGF* and locations of analyzed polymorphisms

The *VEGFA* also known as *VEGF* is located at 6p21.3 and it comprises eight exons and seven introns (Fig. 1) [13]. It is highly polymorphic with several polymorphisms in the promoter, 5′-untranslated region (5′-UTR) and 3′-UTR [14, 15]. Polymorphisms in the promoter and UTRs have been reported to regulate VEGF expression via alternative initiation of transcription and internal initiation of translation [16, 17]. Functional genetic polymorphisms which alter the regulation of gene expression are predicted to have a significant impact on disease pathogenesis [18]. *VEGF* −2578C/A, −2549I/D, −460T/C, −116G/A, +405C/G and +936C/T polymorphisms have been associated with differential expression of VEGF [14, 15, 19–22]. The importance of *VEGFA* in breast cancer has been described in several studies [23, 24]. Increased expression of VEGF has been documented in invasive and non invasive breast cancer tissue [25, 26]. Polymorphisms in promoter, 5′-UTR and 3′-UTR of *VEGF* have been reported to affect translation efficiency, circulating plasma concentrations and tumor tissue expression of VEGF [19, 27]. It has been documented that *VEGF* polymorphisms influencing VEGF expression in normal cells might have an impact on tumorigenesis, tumor progression, and response to anti-VEGF agents [22, 28–30].

Haplotype analysis could be a better predictive approach rather than investigating individual polymorphism. It estimates more specific risk and reduces the dimension of association tests and increase statistical power [31]. Due to the important role of *VEGF* in carcinogenesis, the present study aimed to investigate the association of haplotypes of *VEGF* −2578C/A (−1540C/A), −2549I/D (−1511I/D), −460T/C (−1498T/C), +405C/G (−634C/G), −7C/T (+1032C/T) and +936C/T polymorphisms with breast cancer risk in North-West Indians. So far there is no combined report on these six *VEGF* polymorphisms in breast cancer. To the best of our knowledge, this is the first study evaluating the potential association of haplotypes of *VEGF* −2578C/A (rs699947), −2549I/D (rs35569394), −460T/C (rs833061), +405C/G (rs2010963) −7C/T (rs25648) and +936C/T (rs3025039) polymorphism with breast cancer risk.

## Methods

### Subjects

The study was performed according to Declaration of Helsinki and was approved by the Ethics Committee of Guru Nanak Dev University, Amritsar, Punjab, India. All the subjects gave a written informed consent with a signature or thumb impression. A total of 500 subjects (250 breast cancer patients and 250 healthy controls) were analyzed in this study. The patients were investigated at Sri Guru Ram Das Institute of Medical Sciences and Research, Vallah, Amritsar, Punjab (India). The selection criteria of patients and controls have been described in our previous study [32]. All the subjects gave 5 ml blood samples for genetic analyses.

### Genotyping of *VEGF* polymorphisms and analyses of data

The DNA was extracted from EDTA-anti-coagulated blood samples using organic method [33] with few modifications. Three promoter (*VEGF* −2578C/A, −2549I/D, −460T/C), two 5′-UTR (+405C/G, −7C/T), and one 3´-UTR (+936C/T) polymorphisms were analyzed in this study (Fig. 1). The *VEGF* −2549I/D polymorphism was analyzed by direct PCR. *VEGF* −460T/C, −2578C/A +405C/G and *VEGF* +936C/T polymorphisms of *VEGF* were analyzed using PCR–RFLP method. *VEGF* −7C/T polymorphism was analyzed by ARMS-PCR. Ten percent of randomly selected samples were sequenced to validate the PCR based assay genotyping and results of both sets of analyses were 100% concordant. The detail of reaction conditions and analysis of genotype data have been described in our published studies [34, 35]. To determine the better contribution of *VEGF* polymorphisms to breast cancer risk, haplotypes of six *VEGF* polymorphisms were generated using the online SNPStats software [36]. Further we predicted the possible influence of studied *VEGF* polymorphisms on the transcription factor binding sites using online software TFSEARCH (http://www.cbrc.jp/research/db/TFSEARCH.html).

## Results

### Characteristics of study participants

The demographic characteristics of study participants were presented in Table 1. The mean age of patients was 49.38 ± 11.87 years and of controls was 49.34 ± 11.85 years. Of the 250 breast cancer patients, 234 (93%) had infiltrating ductal carcinoma, 4 (2%) had infiltrating lobular carcinoma and 12 (5%) had other types of cancer like medullary carcinoma, mucinous carcinoma, Paget’s disease and phyllodes tumor. In the present study, 127 (51%) cases had tumor in left breast, 112 (45%) in right breast and 11 (4%) cases had tumor in both breasts. Of the 250 breast cancer patients, 65 (26%) had stage I, 119 (48%) had stage II, 48 (19%) had stage III, and 18 (7%) had stage IV tumor.

**Table 1:** Characteristics of Breast cancer patients and healthy controls

Variable	Patients n	%	Controls n	%
Total No. of subjects	250		250	
Sex				
Males	7	2.8	7	2.8
Females	243	97.2	243	97.2
Age at diagnosis (years)				
≤ 40	66	26.4	66	26.4
> 40	184	73.6	184	73.6
Mean age	49.38 ± 11.87		49.34 ± 11.85	
Range	25–85		25–85	
Habitat				
Rural	186	74.4	186	74.4
Urban	64	25.6	64	25.6
Diet				
Vegetarian	147	58.8	154	61.6
Non vegetarian	103	41.2	96	38.4
Obesity				
Non obese	62	24.8	70	28.0
Obese	188	75.2	180	72.0
Menstrual status				
Premenopausal	114	46.91	150	61.73
Postmenopausal	129	53.09	93	38.27
Mean age at menarche	14.74 ± 1.78		14.75 ± 1.47	
Mean age at first child birth	22.70 ± 4.08		22.45 ± 3.43	
Breastfeeding				
Yes	215	88.48	224	92.18
No	28	11.52	19	7.82
Oral Contraceptives				
Yes	21	8.64	16	6.58
No	222	91.36	227	93.42

### Association of *VEGF* polymorphisms with breast cancer

The results of association of individual *VEGF* polymorphism were summarized (Additional file 1: Table S1). The AA genotype and A allele of *VEGF* −2578C/A, II genotype and I allele of *VEGF* −2549I/D, CC genotype and C allele of *VEGF* −460T/C, GG genotype and G allele of *VEGF* +405C/G polymorphism was significantly associated with increased risk of breast cancer. No association of *VEGF* −7C/T and +936C/T polymorphism with breast cancer risk was observed. We analyzed haplotypes of *VEGF* −2578C/A, −2549I/D, −460T/C, +405C/G, −7C/T and +936C/T polymorphisms to determine if there is any difference in *VEGF* haplotypes between breast cancer patients and healthy controls. The most common haplotype in the present study was AICGCC, with the frequencies of 31.3% in breast cancer patients and 23.8% in healthy control individuals. We observed that CDTCCC (OR = 0.56, 95%CI, 0.38–0.81; *p* = 0.003) and CDTGCC (OR = 0.63, 95%CI, 0.44–0.92; *p* = 0.018) haplotypes were significantly associated with decreased risk of breast cancer (Table 2). CDTCCC haplotype was significantly associated with reduced risk of breast cancer in pre and post menopausal patients (Tables 3 and 4). None of the *VEGF* haplotype was associated with breast cancer risk in pre menopausal patients when compared with post menopausal patients (Table 5). The CDTCCC haplotype was also significantly associated with decreased risk of breast cancer in both obese and non obese patients (Tables 6 and 7). Haplotype CDTGCC was marginally associated (*p* = 0.07) with reduced risk of breast cancer in non-obese patients as compared with non-obese controls (Table 7) where as haplotype AICGTC was marginally associated (*p* = 0.09) with reduced risk of breast cancer in obese patients when compared with non-obese patients (Table 8). Further we compared pre menopausal obese patients with post menopausal obese patients and observed that CDTGCC haplotype was significantly associated (*p* = 0.02) with increased risk of breast cancer in premenopausal obese patients (Table 9).

**Table 2:** Association between *VEGF* haplotypes and breast cancer risk

Haplotype^a^	Cases (%)	Controls (%)	OR(95%CI)	*p* value
AICGCC	31.3	23.8	1 (Reference)	
CDTCCC	23.7	28.9	0.56 (0.38–0.81)	**0.003**
CDTGCC	19.6	25.6	0.63 (0.44–0.92)	**0.018**
AICGTC	9.0	9.3	0.75 (0.43–1.30)	0.31
AICGCT	3.1	1.8	1.22 (0.40–3.74)	0.73
AICGTT	1.8	2.0	0.76 (0.25–2.33)	0.64
CDTCCT	1.7	1.2	1.25 (0.30–5.19)	0.76
CDTGCT	1.4	1.5	0.65 (0.13–3.35)	0.61

Significant *p* values are shown in bold

*OR* odds ratio, *CI* confidence interval

^a^In order of −2578C/A, −2549I/D, −460T/C, +405C/G, −7C/T and +936C/T

**Table 3:** *VEGF* haplotypes and breast cancer risk in premenopausal subjects

Haplotype^a^	Pre menopausal cases (%) n = 114	Pre menopausal controls (%) n = 150	OR (95%CI)	*p* value
AICGCC	29.0	24.5	1 (Reference)	
CDTCCC	20.6	28.5	0.65 (0.43–0.99)	**0.04**
CDTGCC	25.0	26.3	0.95 (0.64–1.42)	0.81
AICGTC	9.0	9.9	0.90 (0.50–1.64)	0.73
AICGCT	3.1	1.4	2.23 (0.67–7.43)	0.18
AICGTT	3.2	1.5	2.27 (0.69–7.51)	0.16
CDTCCT	3.3	2.1	1.87 (0.46–7.56)	0.38

Significant *p* values are shown in bold

*OR* odds ratio, *CI* confidence interval

^a^In order of −2578C/A, −2549I/D, −460T/C, +405C/G, −7C/T and +936C/T

**Table 4:** *VEGF* haplotype and breast cancer risk in post menopausal subjects

Haplotype^a^	Post menopausal cases (%) n = 129	Post menopausal controls (%) n = 93	OR (95%CI)	*p* value
AICGCC	31.9	23.4	1 (Reference)	
CDTCCC	24.8	33.3	0.52 (0.30–0.92)	**0.02**
CDTGCC	17.6	22.4	0.59 (0.31–1.12)	0.11
AICGTC	9.2	7.9	0.82 (0.34–1.99)	0.66
AICGCT	3.3	2.4	1.05 (0.16–6.83)	0.96
AICGTT	0.7	3.3	0.27 (0.04–1.94)	0.19

Significant *p* values are shown in bold

*OR* odds ratio, *CI* confidence interval

^a^In order of −2578C/A, −2549I/D, −460T/C, +405C/G, −7C/T and +936C/T

**Table 5:** Association of *VEGF* haplotypes with breast cancer risk in pre menopausal and post menopausal patients

Haplotype^a^	Pre menopausal (%) n = 114	Post menopausal (%) n = 129	OR (95%CI)	*p* value
AICGCC	29.0	31.9	1 (Reference)	
CDTCCC	20.6	24.8	0.95 (0.54–1.67)	0.86
CDTGCC	25.0	17.6	1.42 (0.82–2.47)	0.22
AICGTC	9.0	9.2	1.05 (0.48–2.33)	0.90
AICGCT	3.1	3.3	0.70 (0.12–3.90)	0.68
CDTCCT	3.8	1.1	3.01 (0.55–16.65)	0.21
AITGTC	2.8	1.8	0.24 (0.03–1.97)	0.18
AITGCC	1.5	1.2	1.31 (0.29–5.96)	0.73

*OR* odds ratio, *CI* confidence interval

^a^In order of −2578C/A, −2549I/D, −460T/C, +405C/G, −7C/T and +936C/T

**Table 6:** Association of *VEGF* haplotypes with breast cancer risk in obese subjects

Haplotype^a^	Obese patients (%) n = 188	Obese controls (%) n = 180	OR (95%CI)	*p* value
AICGCC	31.2	24.6	1 (Reference)	
CDTCCC	25.2	28.9	0.63 (0.41–0.99)	**0.04**
CDTGCC	19.3	25.0	0.76 (0.49–1.19)	0.23
AICGTC	7.0	9.3	0.70 (0.35–1.39)	0.31
AICGTT	3.0	1.9	1.27 (0.39–4.12)	0.69
CDTGCT	2.4	1.7	1.23 (0.29–5.22)	0.78
AICGCT	1.8	1.7	0.77 (0.14–4.26)	0.76
CDTCCT	1.8	1.5	1.17 (0.26–5.18)	0.84

Significant *p* values are shown in bold

*OR* odds ratio, *CI* confidence interval

^a^In order of −2578C/A, −2549I/D, −460T/C, +405C/G, −7C/T and +936C/T

**Table 7:** Association of *VEGF* haplotypes with breast cancer risk in non obese subjects

Haplotype^a^	Non-obese patients (%) n = 62	Non-obese controls (%) n = 70	OR(95%CI)	*p* value
AICGCC	34.1	23.7	1 (Reference)	
CDTCCC	20.9	31.2	0.44 (0.21–0.93)	**0.03**
CDTGCC	18.2	22.7	0.47 (0.21–1.05)	0.07
AICGTC	13.5	9.5	1.11 (0.38–3.21)	0.85
CDTGTC	1.2	1.4	0.46 (0.02–12.99)	0.65

Significant *p* values are shown in bold

*OR* odds ratio, *CI* confidence interval

^a^In order of −2578C/A, −2549I/D, −460T/C, +405C/G, −7C/T and +936C/T

**Table 8:** Association of *VEGF* haplotypes with breast cancer risk in obese and non obese patients

Haplotype^a^	Obese (%) n = 188	Non-obese (%) n = 62	OR (95%CI)	*p* value
AICGCC	31.2	34.1	1 (Reference)	
CDTCCC	25.1	20.9	1.28 (0.70–2.34)	0.42
CDTGCC	19.3	18.2	1.11 (0.61–2.03)	0.73
AICGTC	7.0	13.5	0.48 (0.21–1.12)	0.09
CDTGTC	3.1	1.2	2.74 (0.33–22.40)	0.35
AICGCT	1.8	4.8	0.27 (0.04–1.91)	0.19

*OR* odds ratio, *CI* confidence interval

^a^In order of −2578C/A, −2549I/D, −460T/C, +405C/G, −7C/T and +936C/T

**Table 9:** Association of *VEGF* haplotypes with breast cancer risk in pre menopausal obese and post menopausal obese patients

Haplotype^a^	Pre menopausal obese (%) n = 85	Post menopausal obese (%) n = 102	OR (95%CI)	*p* value
AICGCC	25.0	35.1	1 (Reference)	
CDTCCC	21.8	25.5	1.13(0.60–2.13)	0.7
CDTGCC	27.6	15.0	1.98(1.10–3.56)	**0.02**
CDTGCT	1.8	2.7	1.45(0.27–7.71)	0.66
AICGTC	8.6	7.9	1.27(0.50–3.24)	0.62

Significant *p* values are shown in bold

*OR* odds ratio, *CI* confidence interval

^a^In order of −2578C/A, −2549I/D, −460T/C, +405C/G, −7C/T and +936C/T

The TFSEARCH software was used to predict the functional significance of *VEGF* polymorphisms. Based on the difference in TFSEARCH TFBS scores, *VEGF* −2578C/A and +405C/G polymorphisms were predicted to alter a transcription factor binding site. *VEGF* −2578A allele abolish the binding site of GATA-2 transcription factor where as *VEGF* +405G allele created the binding site of MZF1 (Myeloid zinc finger 1) transcription factor.

## Discussion

In the present study we investigated the potential association of *VEGF* haplotypes based on six polymorphisms with breast cancer risk. In previous reported studies, by using the single/double or triple polymorphism approach, *VEGF* −2578C/A, −2549I/D, −460T/C, +405C/G, −7C/T and +936C/T polymorphisms have been analyzed to evaluate their potential association with breast cancer risk in different ethnic groups and results are conflicting (Additional file 1: Table S2). The ethnicity difference and inadequate sample size could be the potential cause of inconsistent results.

In the present study, we observed that CDTCCC (OR = 0.56, 95%CI, 0.38–0.81; *p* = 0.003) and CDTGCC (OR = 0.63, 95%CI, 0.44–0.92; *p* = 0.018) haplotypes of *VEGF* −2578C/A, −2549I/D, −460T/C, +405C/G, −7C/T and +936C/T polymorphisms were significantly associated with reduced risk of breast cancer. In none of the previous studies, these six polymorphisms have been reported together. In Caucasian subjects, − 460T/+405C/ − 7C/, +936C haplotype was associated with reduced risk of breast cancer [46]*.* Significant association of *VEGF* −2578A/−1154A/+405G haplotype with decreased risk of invasive breast cancer has been reported in American population [44]. Haplotype *VEGF* −1154A/−2578A/−634G/−460C was associated with decreased risk of breast cancer in Moroccan population [39]. The −2578A/−1154G/+405G haplotype was associated with decreased risk whereas haplotype −2578C/−1154G/+405G was associated with increased risk of breast cancer recurrence in Caucasian women [58]. Association of −2578C/+405C haplotype with tumor size and higher histological grade has been documented in breast cancer patients [45]. None of the haplotype of *VEGF* −2578C/A, −2549I/D, −460T/C, +405C/G, and +936C/T polymorphisms was associated with breast cancer risk in Iranian population [38].

*VEGF* −460C/+405G/+936T haplotype was associated with decreased risk of lung cancer in Koreans [59] and increased risk of esophageal adenocarcinoma in Caucasian [60]. The TGC haplotype of *VEGF* −460C/T, +405C/G and +936C/T polymorphism was significantly associated with decreased risk of adenocarcinoma among male non-small cell lung cancer patients [61]. In Turkish population, *VEGF* −2578A/+936T/−460T haplotype has been reported to be associated with increased risk of colorectal cancer [62]. In Tunisians, CIC haplotype of *VEGF* −2578C/A, −2549I/D and +936C/T polymorphisms was associated with increased risk of urothelial bladder cancer [63].

There are some studies from India on different diseases showing association of *VEGF* haplotypes with disease risk. The CTIG haplotype of *VEGF* −2578C/A, −7C/T, −2549I/D, and −1001G/C polymorphisms was associated with increased risk of bladder cancer [64] whereas TACI haplotype of *VEGF* +936C/T, −1154G/A, −2578C/A and −2549I/D polymorphisms was associated with increased risk of end stage renal disease [65]. Haplotypes CGCC and CGGC of *VEGF* −460T/C, −1154G/A, +405C/G, and +936C/T polymorphism were associated with aggressiveness of disease in epithelial ovarian cancer patients [66]. No association of *VEGF* +405C/G and +936C/T haplotypes with lung cancer risk has been reported in Kashmiri patients [67].

In the present study, CDTCCC haplotype was significantly associated with reduced risk of breast cancer in pre menopausal as well as in post menopausal patients when compared with pre and post menopausal controls. The breast cancer risk has also been reported to be modulated by menopause [68]. Estrogen exposure has been described as an important risk factor for breast cancer development and progression [69]. It has been documented that estrogen modulates angiogenesis via effects on endothelial cells under both physiologic and pathologic conditions [70]. Association of *VEGF* −460T/+405G/+936T haplotype with reduced risk of breast cancer has been reported in Chinese premenopausal women [47]. Among post-menopausal breast cancer patients, CCCCC haplotype of *VEGF* −2578C/A, −2489C/T, −460T/C, +405C/G and −7C/T polymorphisms was associated with reduced risk of distant metastases [71].

The CDTCCC haplotype was significantly associated with decrease risk of breast cancer in obese as well as in non obese patients compared to obese and non obese controls where as CDTGCC haplotype was significantly associated with increased risk of breast cancer in premenopausal obese patients. About 75.2% of patients and 72% of controls were obese in the present study. It has been hypothesized that hormonal mechanisms and metabolic factors are involved in the link between obesity and breast cancer. Insulin resistance and hyperinsulinemia have been reported to be associated with increased breast cancer risk and with worst prognosis in both pre and post menopausal women [72–74]. In mouse model, it has been demonstrated that over expression of VEGFA in adipose tissue provide protection against high fat diet induced obesity and insulin sensitivity [75, 76]. It has been documented that angiogenesis plays an important role in the regulation of adipogenesis [77]. *VEGF* has been described as an important angiogenic factor in adipose tissue and it regulates the development of new vessels required for the expansion of adipose tissue [76, 78]. It has been reported that adiponectin, a regulator of insulin resistance block angiogenesis by increasing the expression of *TP53* and decreasing the expression of *VEGF* [79].

In the present study we predicted that *VEGF* −2578A allele of *VEGF* −2578C/A polymorphism abolished the binding site of GATA-2 transcription factor. The GATA family of transcription factors is regulator of gene expression in hematopoietic cells [80, 81]. Correlation of reduced GATA binding promoter activity has been documented with attenuation of VEGF mediated signaling [82]. G allele of *VEGF* +405C/G polymorphism created the binding site of MZF1 transcription factor. MZF1 transcription factor has been reported to be involved in transcriptional regulation during myelopoiesis [83]. Disruption of MZF1 transcription factor binding site by *VEGF*-634C (+405C) allele has also been reported in peripheral blood mononuclear cells [15]. It has been reported that substitution of C by G at +405 position in 5′-UTR may affect internal ribosome entry site (IRES) and increases the transcription of large isoform of VEGFA [84].

Polymorphisms of *VEGFA* have been reported to be associated with efficacy and toxicity of anti—VEGF agents [41, 85, 86]. Haplotype −460T/+405C/+936C haplotype was associated with better survival among Chinese breast cancer patients [87]. *VEGF* −2578A/−1154G/+405G haplotype was associated with marginally improved prognosis whereas haplotype −2578C/−1154G/+405G was significantly associated with adverse prognosis in HER2 positive breast cancer patients [88]. Apart from breast cancer, correlation of *VEGF* haplotypes with therapy response has also been documented in other cancer types. The CACC haplotype of *VEGF* −460T/C, −116G/A, +405C/G, and +936C/T polymorphism was significantly associated with worse survival in Korean gastric cancer patients [89]. In esophageal cancer, CGC haplotype of *VEGF* −460T/C, +405C/G and +936C/T polymorphism was associated with poorer outcome as compared to other haplotypes [90]. The AGCGC haplotype of *VEGF* −2578 C/A, −1154 G/A, −460T/C, +405 G/C and +936C/T polymorphisms was found to be associated with improved progression-free survival in epithelial ovarian cancer patients [91]. Haplotype −2578C/−460T/+405C/+936C and −2578C/−460T/+405C/+936T was associated with inferior response rate in metastatic colorectal cancer patients to first line XELOX treatment [92]. Thus, assessment of haplotypes of *VEGF* polymorphisms may have implications for aggressiveness and selection of patients suitable for anti-VEGF therapy in context of previously reported literature. The *VEGF* haplotypes in independent cohorts are insightful for identification of cancer risk.

## Conclusions

We report for the first time that CDTCCC and CDTGCC haplotypes of *VEGF* −2578C/A, −2549I/D, −460T/C, +405C/G, −7C/T and +936C/T polymorphisms were significantly associated with breast cancer risk in North-West Indians. Further studies on multiethnic groups with larger sample size are required to confirm our results.

## Supplementary Information

**Additional file 1.** In silico pathway analysis based on chromosomal instability in breast cancer patients.

## Data Availability

All data generated in this study is included in Additional file 1: Table S1.

## Abbreviations

VEGF: Vascular endothelial growth factor
UTR: Untranslated region
OR: Odds ratio
