# Supplementary material for: Association of VEGF haplotypes with breast cancer risk in North-West Indians
Source: BMC Med Genomics. 2021 Aug 24;14:209. doi: 10.1186/s12920-021-01060-4 (PMC8386001; doi:10.1186/s12920-021-01060-4)
Supplement: Supplementary file 1 — Additional file 1. In silico pathway analysis based on chromosomal instability in breast cancer patients. [file 12920_2021_1060_MOESM1_ESM.docx]

**Table S1. Association of *VEGF* Polymorphisms and Breast cancer risk**

| ***VEGF* -2578C/A polymorphism HWE**: Patients (p = 0.16); Controls (p = 0.18); Both (p = 0.16) | | | | | | | | |
| --- | --- | --- | --- | --- | --- | --- | --- | --- |
|  | **Patients**  **n(%)** | | **Controls**  **n(%)** | | **OR (95%CI)** | | **p value** | |
| **Genotypes** |  | |  | |  | |  | |
| CC | 69(27.6) | | 88(35.2) | | Reference | |  | |
| CA | 114(45.6) | | 129(51.6) | | 1.13(0.75-1.69) | | 0.56 | |
| AA | 67(26.8) | | 33(13.2) | | 2.59(1.54-4.37) | | **0.0003** | |
| **Genetic Models**  ***Dominant***  (CA+AA vs CC) | 181(72.4) | | 162(64.8) | | 1.42(0.97-2.08 ) | | 0.067 | |
| ***Codominant***  (AA vs CA; CA vs CC) |  | |  | | 1.54(1.19-1.98) | | **0.001** | |
| ***Recessive***  (AA vs CC+CA) |  | |  | | 2.41(1.52-3.82) | | **0.00014** | |
| **Alleles** |  | |  | |  | |  | |
| C | 252(50.4) | | 305(61.0) | | Reference | |  | |
| A | 248(49.6) | | 195(39.0) | | 1.54(1.20-1.98) | | **0.0007** | |
| ***VEGF* -2549I/D polymorphism HWE:** Patients (p = 0.13); Controls (p = 0.14); Both (p = 0.11) | | | | | | | | |
| **Genotypes** | |  | |  | |  | |  |
| DD | | 69(27.6) | | 85(34.0) | | Reference | |  |
| ID | | 113(45.2) | | 131(52.4) | | 1.06(0.71-1.59) | | 0.76 |
| II | | 68(27.2) | | 34(13.6) | | 2.46(1.46-4.14) | | **0.0005** |
| **Genetic Models**  ***Dominant***  (ID+II vs DD) | |  | |  | | 1.35(0.92-1.98) | | 0.121 |
| ***Codominant***  (II vs ID; ID vs DD) | |  | |  | | 1.50(1.16-1.93) | | **0.002** |
| ***Recessive***  (II vs DD+ID) | |  | |  | | 2.37(1.50-3.75) | | **0.00016** |
| **Alleles** | |  | |  | |  | |  |
| D | | 251(50.2) | | 301(60.2) | | Reference | |  |
| I | | 249(49.8) | | 199(39.8) | | 1.5(1.17-1.93) | | **0.0014** |
| ***VEGF* -460T/C polymorphism HWE:** Patients (p = 0.35); Controls (p = 0.03); Both (p = 0.06) | | | | | | | | |
| **Genotypes** | |  | |  | |  | |  |
| TT | | 75(30.0) | | 81(32.4) | | Reference | |  |
| TC | | 117(46.8) | | 137(54.8) | | 0.92(0.62-1.38) | | 0.692 |
| CC | | 58(23.2) | | 32(12.8) | | 1.96(1.15-3.34) | | **0.013** |
| **Genetic Models**  ***Dominant***  (TC+CC vs TT) | |  | |  | | 1.12(0.77-1.63) | | 0.562 |
| ***Codominant***  (CC vs TC; TC vs TT) | |  | |  | | 1.31(1.01-1.70) | | **0.037** |
| ***Recessive***  (CC vs TT+TC) | |  | |  | | 2.06(1.28-3.30) | | **0.002** |
| **Alleles** | |  | |  | |  | |  |
| T | | 267(53.4) | | 299(59.8) | | Reference | |  |
| C | | 233(46.6) | | 201(40.2) | | 1.30(1.01-1.67) | | **0.041** |
| ***VEGF* +405C/G polymorphism HWE:** Patients (p = 0.30); Controls (p = 0.23); Both (p = 0.29) | | | | | | | | |
| **Genotypes** | |  | |  | |  | |  |
| CC | | 15(6.0) | | 25(10.0) | | Reference | |  |
| CG | | 105(42.0) | | 121(48.4) | | 1.45(0.72-2.89) | | 0.294 |
| GG | | 130(52.0) | | 104(41.6) | | 2.08(1.04-4.15) | | **0.034** |
| **Genetic Models**  ***Dominant***  (CG+GG vs CC) | |  | |  | | 1.74(0.89-3.39) | | 0.098 |
| ***Codominant***  (GG vs CG; CG vs CC) | |  | |  | | 1.44(1.09-1.91) | | **0.010** |
| ***Recessive***  (GG vs CC+CG) | |  | |  | | 1.52(1.07-2.17) | | **0.020** |
| **Alleles** | |  | |  | |  | |  |
| C | | 135(27.0) | | 171(34.2) | | Reference | |  |
| G | | 365(73.0) | | 329(65.8) | | 1.59(1.18-2.14) | | **0.013** |
| ***VEGF* -7C/T polymorphism** | | | | | | | | |
| **Genotypes** | |  | |  | |  | |  |
| CC | | 170(68.0) | | 184(73.6) | | Reference | |  |
| CT | | 80(32.0) | | 66(26.4) | | 1.31(0.89-1.93) | | 0.17 |
| TT | | - | | - | |  | |  |
| **Alleles** | |  | |  | |  | |  |
| C | | 420(84.0) | | 434(86.8) | | Reference | |  |
| T | | 80(16.0) | | 66(13.2) | | 1.25(0.88-1.78) | | 0.21 |
| ***VEGF* +936C/T polymorphism HWE**: Patients (p = 0.46); Controls (p = 0.69); Both (p = 0.70) | | | | | | | | |
| **Genotypes** | |  | |  | |  | |  |
| CC | | 207(82.8) | | 213(85.2) | | Reference | |  |
| CT | | 42(16.8) | | 36(14.4) | | 1.20(0.74-1.95) | | 0.46 |
| TT | | 1(0.4) | | 1(0.4) | | 1.03(0.06-16.56) | | 0.98 |
| **Genetic Models**  ***Dominant***  (CT+TT vs CC) | |  | |  | | 1.20(0.74-1.93) | | 0.46 |
| ***Codominant***  (TT vs CT; CT vs CC) | |  | |  | | 1.44(1.09-1.91) | | 0.48 |
| ***Recessive***  (TT vs CC+CT) | |  | |  | | 1.00(0.06-16.08) | | 1 |
| **Alleles** | |  | |  | |  | |  |
| C | | 456(91.2) | | 462(92.4) | | Reference | |  |
| T | | 44(8.8) | | 38(7.6) | | 1.17(0.75-1.84) | | 0.49 |

OR-odds ratio; CI-confidence interval; Significant p values are shown in bold

**Table S2. Summary of reported studies on *VEGF* Polymorphisms in Breast cancer**

| ***VEGF* -2578C/A (-1540C/A) Polymorphism** | | | | | | | | | | | |
| --- | --- | --- | --- | --- | --- | --- | --- | --- | --- | --- | --- |
| **Ethnicity** | **Nationality** | **Cases/**  **Controls** | | **Genotype Distribution** | | **Key Findings** | | | **Reference** | | |
|  |  |  |  | **Patients**  **CC/CA/AA** | **Controls**  **CC/CA/AA** |  |  |  |  |  |  |
| Asian | North India | 250/250 | | 69/114/67 | 88/129/33 | ↑ risk with AA genotype and A allele | | | **Present study** | | |
| Mixed | Saudi Arab | 100/100 | | 37/45/18 | 54/37/9 | ↑ risk with AA genotype and A allele | | | [37] | | |
| Mixed | Iran | 250/215 | | 76/138/36 | 94/100/21 | ↑ risk with A allele | | | [38] | | |
| African | Morocco | 70/70 | | 32/28/10 | 19/40/11 | ↓ risk with A allele | | | [39] | | |
| Asia | Thailand | 483/414 | | 240/213/30 | 214/173/27 | No association | | | [40] | | |
| Mixed | America | 520/715 | | - | - | ↑ risk with AA genotype | | | [41] | | |
| Mixed | Austria | 804/804 | | - | - | No association | | | [42] | | |
| Caucasian | England | 2015/2139 | | 498/1012/505 | 544/1054/541 | No association | | | [43] | | |
| Caucasian | USA | 498/495 | | 139/245/114 | 129/236/130 | ↑ risk with C allele for invasive breast cancer | | | [44] | | |
| Caucasian | Poland | 411/423 | | 104/195/112 | 106/207/110 | No association | | | [45] | | |
|  | Germany | 153/162 | | 44/75/34 | 50/72/40 | No association | | |  |  |  |
|  | Sweden | 939/940 | | 258/449/232 | 257/451/232 | No association | | |  |  |  |
| Caucasian | England | 493/498 | | 111/248/134 | 106/251/141 | No association | | | [46] | | |
| Asian | China | 1123/1222 | | 89/418/616 | 78/479/665 | No association | | | [47] | | |
| ***VEGF* -2549I/D (-1511I/D) Polymorphism** | | | | | | | | | | | |
| **Ethnicity** | **Nationality** | **Cases/**  **Controls** | **Genotype Distribution** | | | | **Key Findings** | | | **Reference** | |
|  |  |  | **Patients**  **DD/ID/II** | | **Controls**  **DD/ID/II** | |  |  |  |  |  |
| Asian | North India | 250/250 | 69/113/68 | | 85/131/34 | | ↑ risk with II genotype and I allele | | | **Present study** | |
| Mixed | Iran | 250/215 | 78/135/37 | | 52/134/29 | | No association | | | [38] | |
| ***VEGF* -460T/C (-1498T/C) Polymorphism** | | | | | | | | | | | |
| **Ethnicity** | **Nationality** | **Cases/**  **Controls** | | **Genotype Distribution** | | | | **Key Findings** | | | **Reference** |
|  |  |  |  | **Patients**  **CC/CT/TT** | **Controls**  **CC/CT/TT** | | |  |  |  |  |
| Asian | North India | 250/250 | | 58/117/75 | 32/137/81 | | | ↑ risk with CC genotype and C allele | | | **Present study** |
| Mixed | Iran | 250/215 | | 33/148/69 | 18/131/66 | | | No association | | | [38] |
| African | Morocco | 70/70 | | 18/25/27 | 15/39/16 | | | ↓ risk with C allele | | | [39] |
| Asian | Thailand | 483/411 | | 26/214/243 | 24/172/215 | | | No association | | | [40] |
| Mixed | America | 520/715 | | - | - | | | ↑ risk with CC genotype | | | [41] |
| Mixed | Austria | 804/804 | | - | - | | | No association | | | [42] |

**Table S2. Contd.**

| ***VEGF* +405C/G (-634C/G) Polymorphism** | | | | | | | | | | | |
| --- | --- | --- | --- | --- | --- | --- | --- | --- | --- | --- | --- |
| **Ethnicity** | **Nationality** | **Cases/**  **Controls** | | **Genotype Distribution** | | | | | **Key Findings** | | **Reference** |
|  |  |  |  | **Cases**  **CC/CG/GG** | | | **Controls**  **CC/CG/GG** | |  |  |  |
| Asian | North India | 250/250 | | 15/105/130 | | | 25/121/104 | | ↑ risk with GG genotype and G allele | | **Present study** |
| Mixed | Iran | 250/215 | | 47/102/101 | | | 36/106/73 | | No association | | [38] |
| African | Morocco | 70/70 | | 11/33/26 | | | 4/30/36 | | No association | | [39] |
| Asian | India | 200/200 | | 23/88/89 | | | 26/89/85 | | No association | | [48] |
| Asian | India | 200/200 | | 89/88/23 | | | 85/89/26 | | No association | | [49] |
| Asian | Thailand | 483/355 | | 61/199/223 | | | 40/81/234 | | ↑ risk with C allele | | [40] |
| Asian | China | 680/680 | | 137/205/338 | | | 135/204/341 | | No association | | [50] |
| Mixed | Brazil | 235/235 | | 38/102/95 | | | 24/129/82 | | ↑ risk with CC genotype | | [51] |
| Mixed | Austria | 804/804 | | - | | | - | | No association | | [42] |
| Mixed | America | 520/715 | | - | | | - | | No association | | [41] |
| Caucasian | England | 490/498 | | 57/207/226 | | | 64/225/209 | | No association | | [46] |
| Caucasian | England | 2044/2169 | | 210/872/962 | | | 245/936/988 | | No association | | [43] |
| Caucasian | USA | 495/500 | | 52/222/221 | | | 47/221/232 | | No association | | [44] |
| Asian | China | 1095/1198 | | 192/508/395 | | | 182/598/418 | | No association | | [47] |
| Caucasian | Sweden | 936/941 | | 85/363/488 | | | 82/367/492 | | No association | | [45] |
| ***VEGF* -7C/T (+1032C/T) Polymorphism** | | | | | | | | | | | |
| **Ethnicity** | **Nationality** | **Cases/**  **Controls** | **Genotype Distribution** | | | | | **Key Findings** | | **Reference** | |
|  |  |  | **Cases**  **CC/CT/TT** | | **Controls**  **CC/CT/TT** | | |  |  |  |  |
| Asian | North India | 204/204 | 170/80/0 | | 184/66/0 | | | No association | | **Present study** | |
| Mixed | Austria | 804/804 | - | | - | | | No association | | [42] | |
| Caucasian | England | 490/493 | 344/134/12 | | 332/151/10 | | | No association | | [46] | |
| ***VEGF* +936C/T Polymorphism** | | | | | | | | | | | |
| **Ethnicity** | **Nationality** | **Cases/**  **Controls** | | **Genotype Distribution** | | | | **Key Findings** | | **Reference** | |
|  |  |  |  | **Cases**  **CC/CT/TT** | | **Controls**  **CC/CT/TT** | |  |  |  |  |
| Asian | North India | 250/250 | | 207/42/1 | | 213/36/1 | | No association | | **Present study** | |
| Mixed | Iran | 250/215 | | 179/67/4 | | 159/52/4 | | No association | | [38] | |
| Asian | China | 680/680 | | 446/210/24 | | 426/204/50 | | ↓ risk with TT genotype and T allele | | [50] | |
| Caucasian | Austria | 521/801 | | 371/138/12 | | 580/201/20 | | No association | | [52] | |
| Caucasian | Spain | 447/442 | | 366/76/5 | | 332/99/11 | | ↓ risk with CT and combined CT+TT genotypes | | [53] | |
| Mixed | Brazil | 235/235 | | 190/43/2 | | 176/52/7 | | No association | | [51] | |
| Asian | Taiwan | 220/334 | | 155/59/6 | | 211/117/6 | | ↓ risk with CT genotype | | [54] | |
| Mixed | America | 520/715 | | - | | - | | No association | | [41] | |
| Caucasian | Austria | 804/804 | | - | | - | | No association | | [42] | |
| Caucasian | Poland | 232/225 | | 178/48/6 | | 155/64/6 | | ↓ risk with CT+TT genotypes | | [55] | |
| Caucasian | Turkey | 60/60 | | 44/16/0 | | 57/3/0 | | ↑ risk with CT genotype | | [56] | |
| Caucasian | England | 2016/2107 | | 1509/468/39 | | 1576/495/36 | | No association | | [43] | |
| Caucasian | England | 848/708 | | 624/204/20 | | 531/165/12 | | No association | | [46] | |
| Asian | China | 1109/1195 | | 744/334/31 | | 793/351/51 | | ↓ risk with TT genotype | | [47] | |
| Caucasian | USA | 488/479 | | 360/122/6 | | 353/118/8 | | ↓ risk with C allele for *in situ* breast cancer | | [44] | |
| Caucasian | Poland | 412/422 | | 298/100/14 | | 297/114/11 | | No association | | [45] | |
|  | German | 153/163 | | 120/31/2 | | 128/31/4 | | No association | |  |  |
|  | Sweden | 924/934 | | 708/204/12 | | 720/203/11 | | No association | |  |  |
| Caucasian | Austria | 500/500 | | 412/79/9 | | 353/137/10 | | ↓ risk with CT+TT genotypes | | [57] | |
